# Tandem mass tag (TMT)-based proteomic analysis of *Cryptosporidium andersoni* oocysts before and after excystation

**DOI:** 10.1186/s13071-021-05113-6

**Published:** 2021-12-18

**Authors:** Dong-Fang Li, Zhao-Hui Cui, Lu-Yang Wang, Kai-Hui Zhang, Le-Tian Cao, Shuang-Jian Zheng, Long-Xian Zhang

**Affiliations:** 1 College of Veterinary Medicine, Henan Agricultural University, Zhengzhou, 450000 Henan Province People’s Republic of China; 2 International Joint Research Laboratory for Zoonotic Diseases of Henan, Zhengzhou, 450000 Henan Province People’s Republic of China

**Keywords:** TMT proteomics, *Cryptosporidium andersoni*, Oocyst, Excystation

## Abstract

**Background:**

*Cryptosporidium andersoni* initiates infection by releasing sporozoites from oocysts through excystation. However, the proteins involved in excystation are unknown. Determining the proteins that participate in the excystation of *C. andersoni* oocysts will increase our understanding of the excystation process.

**Methods:**

*Cryptosporidium andersoni *oocysts were collected and purified from the feces of naturally infected adult cows. Tandem mass tags (TMT), coupled with liquid chromatography–tandem mass spectrometry (LC–MS/MS) proteomic analysis, were used to investigate the proteomic expression profiles of *C. andersoni* oocysts before and after excystation.

**Results:**

Proteomic analysis identified a total of 1586 proteins, of which 17 were differentially expressed proteins (DEPs) upon excystation. These included 10 upregulated and seven downregulated proteins. The 17 proteins had multiple biological functions associated with control of gene expression at the level of transcription and biosynthetic and metabolic processes. Quantitative real-time RT-PCR of eight selected genes validated the proteomic data.

**Conclusions:**

This study provides information on the protein composition of *C. andersoni* oocysts as well as possible excystation factors. The data may be useful in identifying genes for diagnosis, vaccine development, and immunotherapy for *Cryptosporidium*.

**Graphical Abstract:**

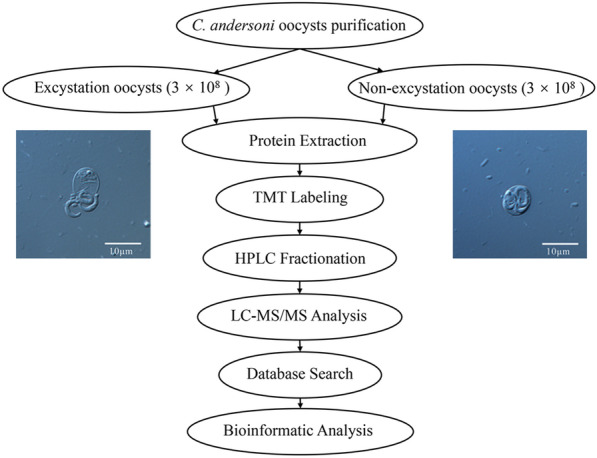

**Supplementary Information:**

The online version contains supplementary material available at 10.1186/s13071-021-05113-6.

## Background

*Cryptosporidium* is an obligate gastrointestinal apicomplexan protozoan parasite that infects all terrestrial vertebrates, including humans, and most aquatic animals [1]. Immunocompromised individuals, particularly patients with acquired immune deficiency syndrome (AIDS) with a late diagnosis or without access to highly active antiretroviral therapy, are very susceptible to *Cryptosporidium* infection [2]. The parasites also cause both sporadic episodes of illness and far-reaching food-borne or waterborne outbreaks [3]. There are no effective treatments or vaccines for cryptosporidiosis at present [2]. Nitazoxanide has been approved by the United States Food and Drug Administration (FDA) as a treatment drug for cryptosporidiosis, but it is not effective in vulnerable malnourished children or immunocompromised patients [4]. The bovine parasite *Cryptosporidium andersoni* was previously considered to be a larger form of *Cryptosporidium muris* based on oocyst size [5]. In 2000, *C. andersoni* was recognized as a new *Cryptosporidium* species based on morphological, biological, and molecular evidence [6]. *Cryptosporidium andersoni* infection can cause weight loss, reduced feed utilization rate, and decreased milk yield in cows. These symptoms seriously impact the success of animal husbandry [7, 8]. *Cryptosporidium andersoni* is the dominant species infecting weaned calves and adult cattle in China [9].

The life-cycle of *Cryptosporidium* begins with the ingestion of oocysts by a susceptible host. The oocysts travel through the digestive tract, excyst in the gastrointestinal tract, and release infective sporozoites that invade the epithelia of the stomach or small intestine [1]. Within host cells, *Cryptosporidium* undergoes successive rounds of asexual and sexual reproduction, where new oocysts are formed and shed with the feces [10]. The mechanism of excystation, a key step in the infection of *Cryptosporidium*, is poorly understood. *Cryptosporidium parvum* has been studied using an in vitro excystation method that simulated the signals sent by the host, including temperature (37 °C), pH, and the presence of cholate, reducing agents, and proteases [11, 12]. Unlike *C. parvum*, the excystation of *C. andersoni* oocysts requires only a single temperature stimulus, indicating that the factors mediating *C. andersoni* excystation may be less complex [13].

The proteomics of *C. parvum* during excystation have been analyzed, and this has increased our understanding of *Cryptosporidium* biology [14]. However, it is not known whether a similar response is solicited when excystation occurs in other species such as *C. andersoni* infecting the abomasum of cattle [6]. In this study, we performed tandem mass tag (TMT)-based proteomic analysis of non-excysted and excysted *C. andersoni* oocysts in order to gain a better understanding of the excystation process.

## Methods

### *Cryptosporidium andersoni* oocyst preparation and excystation

*Cryptosporidium andersoni* oocysts were collected and purified from the feces of naturally infected adult cows (without other pathogenic microorganisms) according to previous reports with appropriate modifications [15]. Briefly, feces of cows were washed three times with phosphate-buffered saline (PBS), and a preliminary purification using Sheather’s sugar flotation method was used to remove impurities (incompletely digested silage), followed by further purification using cesium chloride gradient centrifugation to obtain pure oocysts. Purified *C. andersoni* oocysts were counted using a hemocytometer and stored at 4 °C in PBS (pH 7.2) for no longer than 14 days.

For proteomic analysis, a total of 1.8 × 10^9^ *C. andersoni* oocysts were equally split into non-excysted and excysted groups (9 × 10^8^ oocysts/group). Each group contained three biological replicates (3 × 10^8^ each). Before the experiments, oocysts were subjected to the treatment in 2.5% sodium hypochlorite solution for 10 min at 4 °C and washed three times with PBS. Excystation was performed at 37 °C (~ 3 h and mixing every 10 min) until > 80% excystation was observed by microscopic examination [13].

The morphology of oocysts and sporozoites was also examined using differential interference contrast (DIC) microscopy (Olympus BL53, Tokyo, Japan) and scanning electron microscopy (SEM). For SEM, specimens were fixed overnight at 4 °C in 2.5% glutaraldehyde in 0.1 M phosphate buffer and then washed twice for 15 min with the same buffer. After dehydration in a graded ethanol series, the ethanol was replaced with isoamyl acetate twice for 20 min, dried using the critical point technique, and coated with gold according to a standard protocol. Specimens were examined under a Hitachi S-3400N scanning electron microscope (Tokyo, Japan).

### Protein extraction and SDS-PAGE

Excysted or non-excysted oocysts were suspended in a protein lysis buffer (pH 8.5) containing 7 M urea, 2 M thiourea, 65 mM Tris, 2% dithiothreitol (DTT), 4% 3-[(3-cholamidopropyl)dimethylammonio]-1-propanesulfonate (CHAPS), 0.2% IPG buffer (GE Amersham, Boston, USA), and 0.1% v/v protease inhibitor cocktail (Merck, Darmstadt, Germany) [16]. Samples were disrupted by sonication at 80 W for 3 s × 100 at intervals of 10 s. The debris was removed by centrifugation at 12,000×*g* at 4 °C for 10 min. The supernatants were collected and transferred to new centrifuge tubes. After that, protein concentrations were determined using a BCA Protein Assay Kit (Beyotime Biotechnology, Nanjing, China) according to the manufacturer’s instructions. The quality of protein extracts was evaluated by 12% sodium dodecyl sulfate polyacrylamide gel electrophoresis (SDS-PAGE) fractionation, followed by Coomassie blue staining.

### Trypsin digestion, TMT labeling, and high-performance liquid chromatography fractionation

TMT tagging and analysis were performed as previously described [15]. For protein digestion, the protein extracts were first reduced with 5 mM dithiothreitol for 30 min at 56 °C and alkylated with 11 mM iodoacetamide for 15 min at room temperature in darkness. Samples were diluted by adding 100 mM triethylammonium bicarbonate (TEAB) to urea at a concentration less than 2 M. Trypsin was then added at a 1:50 trypsin-to-protein mass ratio for the first digestion overnight and at a 1:100 trypsin-to-protein mass ratio for a second digestion for 4 h. After digestion, samples were desalted using a Strata-X C18 SPE column (Phenomenex) and vacuum-dried. Peptides were reconstituted in 0.5 M TEAB and processed according to the manufacturer’s protocol for the TMT kit (Thermo Fisher, Waltham, MA, USA). Briefly, one unit of TMT reagent was thawed and reconstituted in acetonitrile. The peptide mixtures were then incubated for 2 h at room temperature and pooled, desalted, and dried by vacuum centrifugation. The tryptic peptides were fractionated by high-pH reversed-phase high-performance liquid chromatography (HPLC) using an Agilent 300 Extend C18 column (5-μm particles, 4.6 mm I.D., 250 mm in length). Peptides were separated into 60 fractions with a gradient of 8–32% acetonitrile (pH 9.0) over 60 min, combined into 18 fractions, and dried by vacuum centrifugation.

### LC–MS/MS analysis and database search

The tryptic peptides were dissolved in 0.1% formic acid (solvent A) and loaded onto a homemade reversed-phase analytical column (15-cm length, 75 µm I.D.). The gradient comprised an increase from 6 to 23% solvent B (0.1% formic acid in 98% acetonitrile) over 26 min, 23%–35% over 8 min, increasing to 80% in 3 min, and then holding at 80% for the final 3 min. All operation procedures occurred at a constant flow rate of 400 nl/min on an EASY-nLC 1000 ultrahigh-performance LC system [17].

The peptides were subjected to a nanospray ionization (NSI) source, followed by tandem mass spectrometry (MS/MS) in a Q Exactive™ Plus system (Thermo Fisher, Waltham, MA, USA) coupled online to the UPLC. The electrospray voltage applied was 2.0 kV. The *m*/*z* scan range was 350–1800 for a full scan, and intact peptides were detected in the Orbitrap at a resolution of 70,000. Peptides were then selected for MS/MS using the NCE setting of 28. The fragments were detected in the Orbitrap at a resolution of 17,500. A data-dependent procedure that alternated between one MS scan followed by 20 MS/MS scans with 15.0-s dynamic exclusion was used. Automatic gain control (AGC) was set at 5E4. The fixed first mass was set as 100 *m*/*z* [18].

The resulting MS/MS data were processed using the MaxQuant search engine (v.1.5.2.8). Tandem mass spectra were searched against the *Cryptosporidium* proteome database (28,217 sequences) from the UniProt database (http://beta.uniprot.org/) concatenated with the reverse decoy database. Trypsin/P was specified as a cleavage enzyme, allowing up to two missing cleavages. The mass tolerance for precursor ions was set as 20 ppm in the first search and 5 ppm in the main search, and the mass tolerance for fragment ions was set as 0.02 Da. Carbamidomethyl on Cys was specified as a fixed modification, and oxidation on Met was specified as a variable modification. The false discovery rate (FDR) was adjusted to < 1.0%, and the minimum score for peptides was set as > 40.

### Bioinformatics analysis

Gene Ontology (GO) annotation of the proteome was derived from the UniProt-GOA database (http://www.ebi.ac.uk/GOA/) that classified proteins into three categories: biological process, cellular compartment, and molecular function. The Eukaryotic Orthologous Groups (KOG) database was used for functional classification of differentially expressed proteins (DEPs). The Kyoto Encyclopedia of Genes and Genomes (KEGG) database was used to annotate metabolic pathways. For GO, KOG, and KEGG enrichment analyses, a two-tailed Fisher’s exact test was applied to test DEPs against all identified proteins, and a corrected *p*-value < 0.05 was considered significant. Identified protein domain functional descriptions were annotated by InterProScan based on the protein sequence alignment method (http://www.ebi.ac.uk/interpro/). The WoLF PSORT program was used to predict subcellular localization (https://www.genscript.com/wolf-psort.html), using an updated version of PSORT/PSORT II for the prediction of eukaryotic sequences.

### Validation of differentially expressed genes by quantitative real-time polymerase chain reaction

Quantitative real-time polymerase chain reaction (qRT-PCR) was used to verify gene expression levels of eight DEPs in non-excysted and excysted *C. andersoni* oocysts [19]. Total RNA of each sample was extracted from excysted and non-excysted oocysts (three biological replicates) using TRIzol™ Reagent (Thermo Fisher, Waltham, MA, USA). RNA purification and reverse transcription were performed using the Reverse Transcriptase M-MLV Kit with gDNA Eraser (Takara, Japan) according to manufacturer’s instructions. The quantity of RNA was analyzed using a NanoDrop One spectrophotometer (Thermo Fisher, Waltham, MA, USA). For each sample, 1 µg of total RNA was treated with 1 µl of gDNA Eraser at 42 °C for 2 min. First-strand complementary DNA (cDNA) was synthesized using 1 µl of Oliga (dT) and 1 µl of Random Primers and RNase-free deionized H_2_O (up to 10 µl) at 70 °C for 10 min and at 4 °C for 2 min. Second-strand cDNA was synthesized using 4 µl of 5× M-MLV buffer, 1 µl of dNTP, 0.5 µl of RI, 0.5 µl of M-MLV, 4 µl RNase-free dH_2_O, and 10 µl of first-strand cDNA. The resulting products were used as templates for qRT-PCR. Gene-specific qRT-PCR primers were designed with Premier 5.0 software (Premier Biosoft International, Palo Alto, CA, USA). Oligonucleotide sequences of target and reference genes (18S rRNA) for qRT-PCR are listed in Additional file 1: Table S1. Each PCR reaction contained 2 µl of cDNA, 0.4 µM (final concentration) of each primer, and 5 µl of 2× SYBR qPCR mix (Takara, Japan). PCR reactions were performed in duplicate using the qTOWER^3^G IVD system (Analytik Jena AG, Germany) with the following cycles: one cycle for denaturing at 95 °C for 30 s; 40 cycles for amplification at 95 °C for 5 s, 55 °C for 10 s, and 72 °C for 15 s; and one cycle of melting curve analysis. Relative expression levels were normalized with those of 18S rRNA. The 2^−ΔΔCT^ method was used to determine the fold change. GraphPad Prism version 8.0 software (https://www.graphpad.com/) was used to analyze and plot the data.

## Results

### Purification and excystation of *C. andersoni* oocysts

**Fig. 1:**
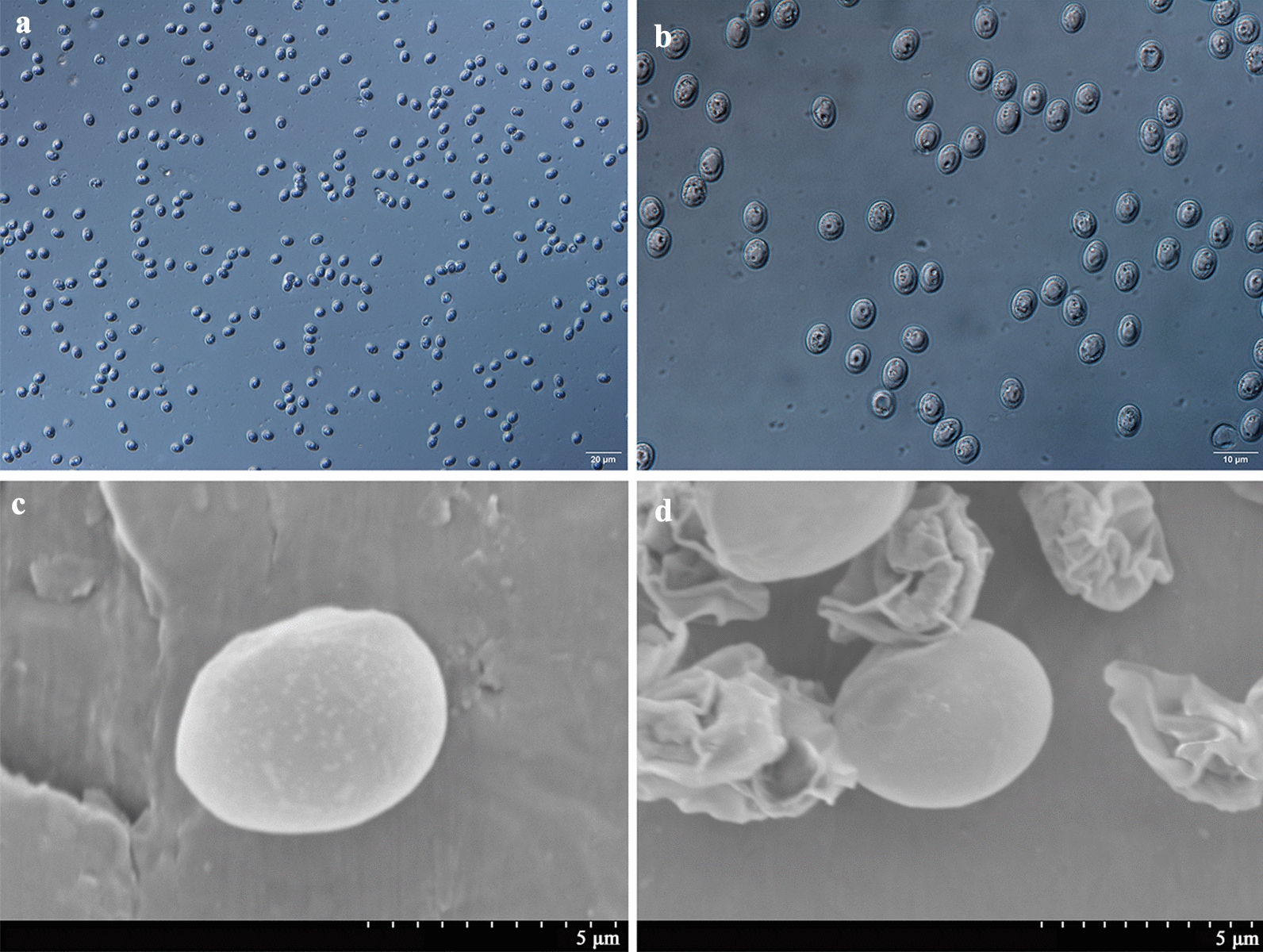
Fresh purified oocysts of *C. andersoni*. **a** and **b** Oocysts of *C. andersoni* were purified from adult cow’s feces with sucrose solution density gradient centrifugation and cesium chloride density gradient centrifugation (**a** ×40; **b** ×100). **c** and **d** Oocysts of *C. andersoni* visualized by SEM

**Fig. 2:**
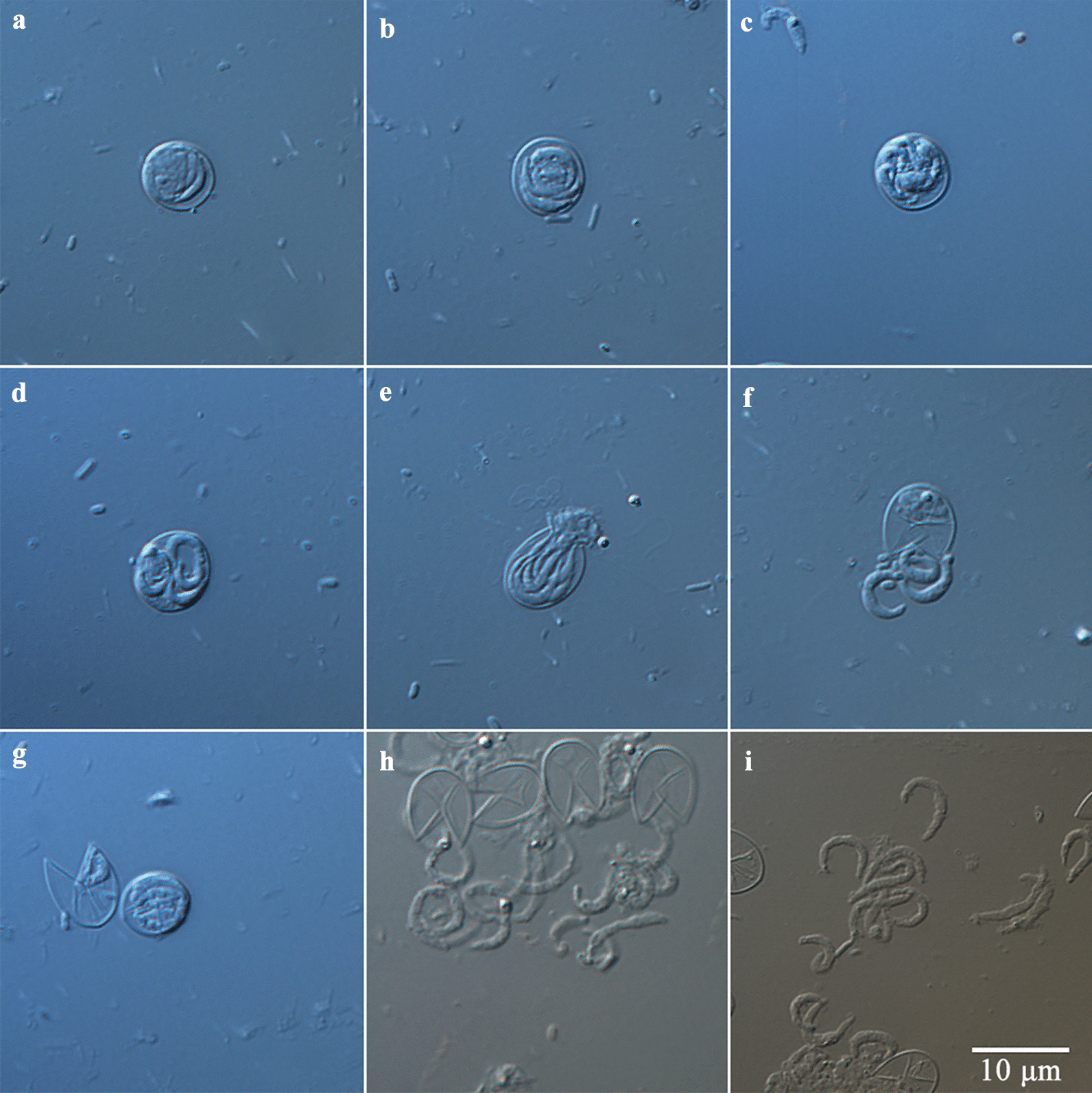
Excystation of *C. andersoni* oocysts. **a**–**d** Non-excysted oocysts. **e** Oocysts ready for excystation. **f** Sporozoite in the process of excystation. **g** Sporozoites remaining inside the oocyst and non-excysted oocysts. **h** Released viable sporozoites and empty oocysts. **i** Released viable sporozoites. Scale bar applies to **a**–**i**

Highly purified oocysts of *C. andersoni* were isolated from the feces of adult cows via sucrose solution density gradient centrifugation and cesium chloride density gradient centrifugation (Fig. 1). The excystation rate of *C. andersoni* was 82% using 3-h incubation at 37 °C. Non-excysted oocysts, excysted oocysts, and sporozoites are shown in Fig. 2. Oocysts were round or oval, with a transparent wall around the oocyst, a bright residual body, and four sporozoites inside the oocyst. The size of oocysts was 7.40 ± 0.55 µm × 5.99 ± 0.49 µm based on the measurement of 100 oocysts. After excystation, the banana-like sporozoites swim snakelike or oscillate violently, and residual bodies either remain in the oocyst or prolapse outside the oocyst wall (Fig. 2i and Additional file 2: Fig. S1).

### Protein identification and quantification

**Fig. 3:**
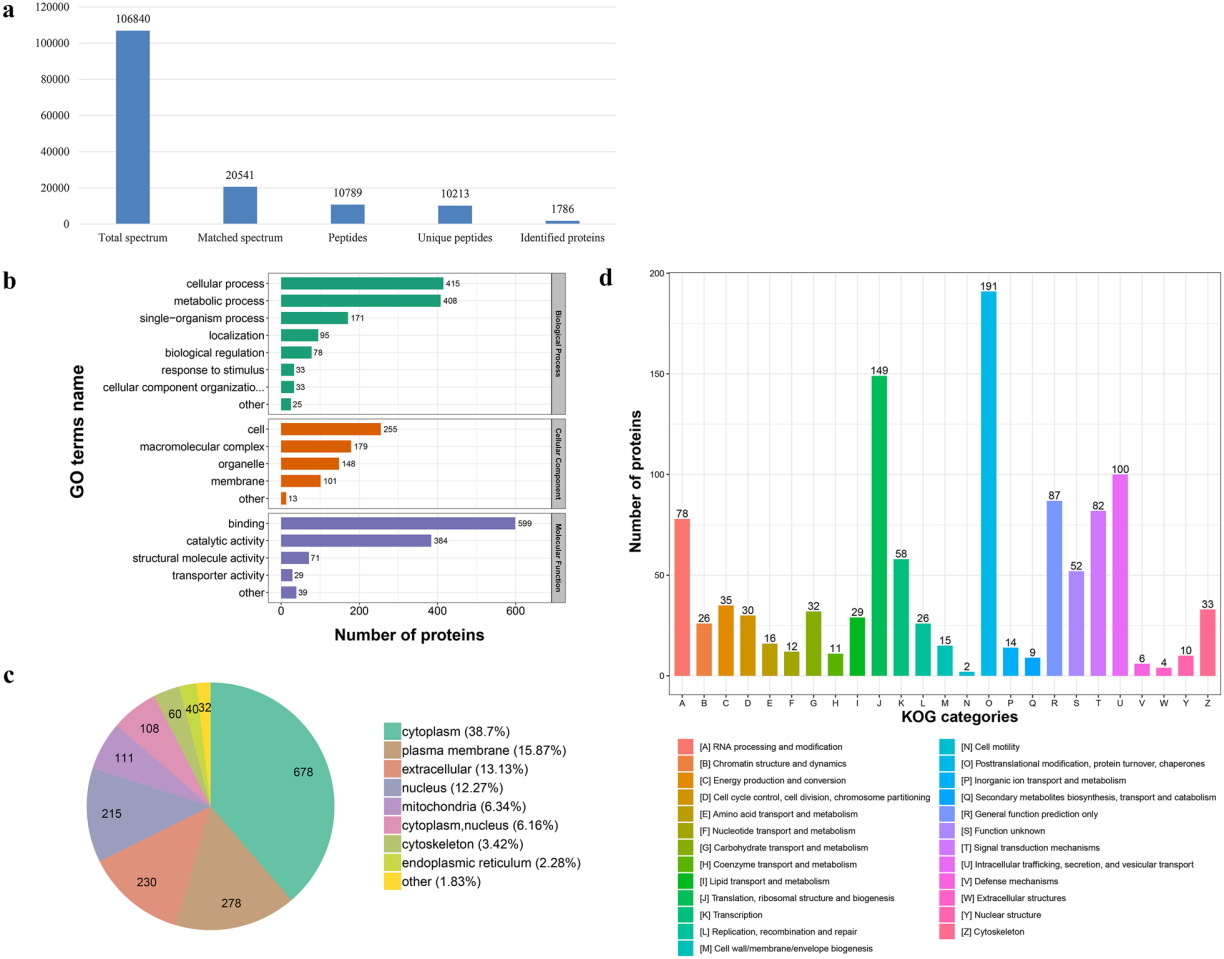
Basic information statistics of identified peptides and bioinformatics analysis of identified proteins. **a** Basic information statistics of identified peptides. **b** Gene Ontology (GO) enrichment of identified proteins. **c** Subcellular localization of identified proteins. **d** KOG enrichment analyses of identified proteins

To understand how the proteome changes with *C. andersoni* oocyst excystation, a TMT-based labeling approach coupled with HPLC and LC–MS/MS was employed to identify the proteome of excysted and non-excysted oocysts. Quality control of protein extraction, labeling efficiency, and mass spectrometry stability showed the methods of mass spectrum analysis are accurate and stable (Additional file 3: Text S1). A total of 106,840 two-stage spectra were obtained by mass spectrometry analysis. A subset of 20,541 effective spectra was obtained after the mass spectrometry two-level graph was searched by the protein theory data, and the spectral graph utilization rate was 19.2%. A total of 10,789 peptide segments were identified by spectral analysis, and 10,213 unique peptide segments and 1786 proteins were identified (Fig. 3). Most of the peptides had 7–20 amino acids, in accordance with the general rules of trypsin-based enzymatic hydrolysis and higher-energy collision-induced dissociation (HCD) fragmentation. In addition, most of the matching errors for the majority of the peptides ranged from −5 to 10 ppm, indicating the good reliability of the TMT data. The statistical analysis of protein identification is provided in Additional file 4: Table S2. The dataset comprised 46.1% of the *C. andersoni* predicted proteome (3876). Among the proteins, 44.3% (791/1786) were uncharacterized proteins, and 4.4% (78/1786) were ribosomal proteins.

### Functional categories of proteins of *C. andersoni* oocysts

**Fig. 4:**
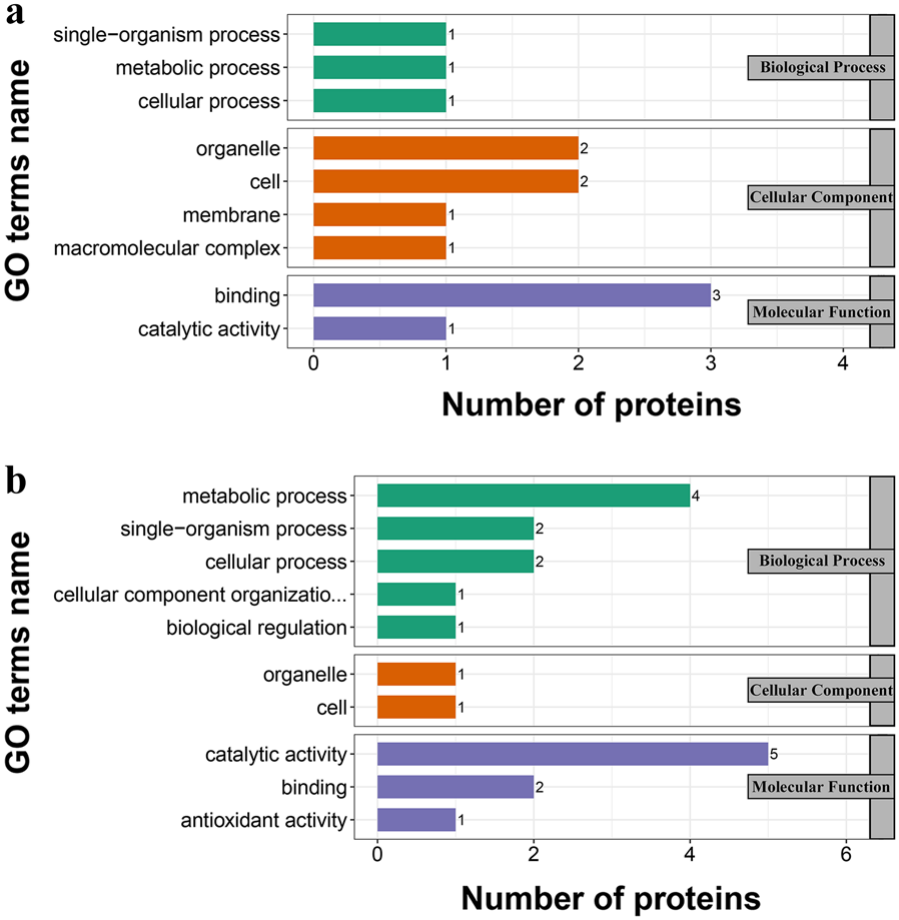
Gene Ontology (GO) enrichment of 17 differentially expressed proteins. **a** GO enrichment of upregulated proteins. **b** GO enrichment of downregulated proteins

All 1786 proteins were subjected to GO, KOG, and subcellular classification analyses (Additional file 4: Table S2). According to the GO analysis, 1258, 696, and 1122 proteins were assigned to the categories “biological processes,” “molecular function,” and “cellular components,” respectively (Fig. 3). The top five most frequent categories of biological processes in this study were “cellular processes” (415), “metabolic processes” (408), “single-organism processes” (171), “localization” (95), and “biological regulation” (78). To predict and classify their possible functions based on reference to the orthologs from other species, all proteins were annotated using the KOG database. Figure 4 shows that a total of 1107 proteins were categorized into 25 groups, among which “posttranslational modification, protein turnover, chaperones” was the largest group (191), followed by “translation, ribosomal structure and biogenesis” (149) and “intracellular trafficking, secretion, and vesicular transport” (100). Subcellular classification showed that those proteins were localized in the “cytoplasm” (678), “plasma membrane” (278), “extracellular space” (230), “nucleus” (215), “mitochondria” (111), “cytoplasm, nucleus” (108), “cytoskeleton” (60), and “endoplasmic reticulum” (40) (Fig. 3).

### TMT analysis and identification of differentially expressed proteins

**Fig. 5:**
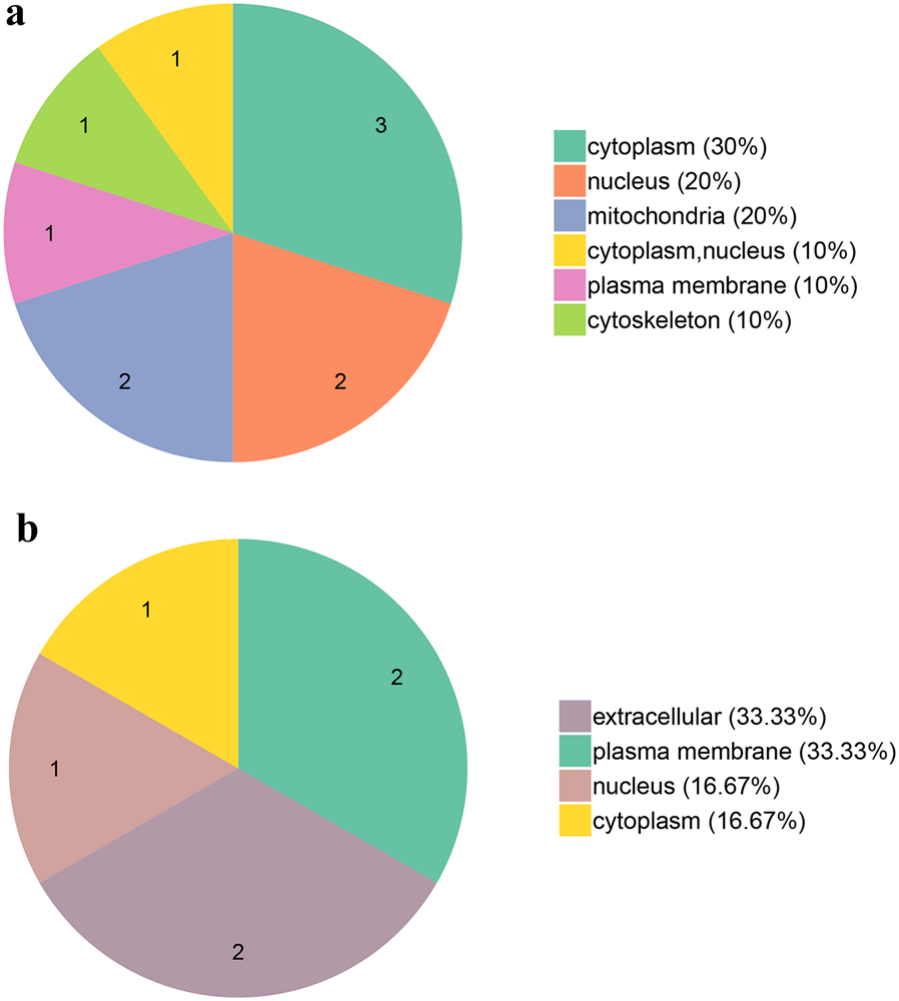
Subcellular localization chart of 17 differentially expressed proteins. **a** Subcellular localization of upregulated proteins. **b** Subcellular localization of downregulated proteins

**Fig. 6:**
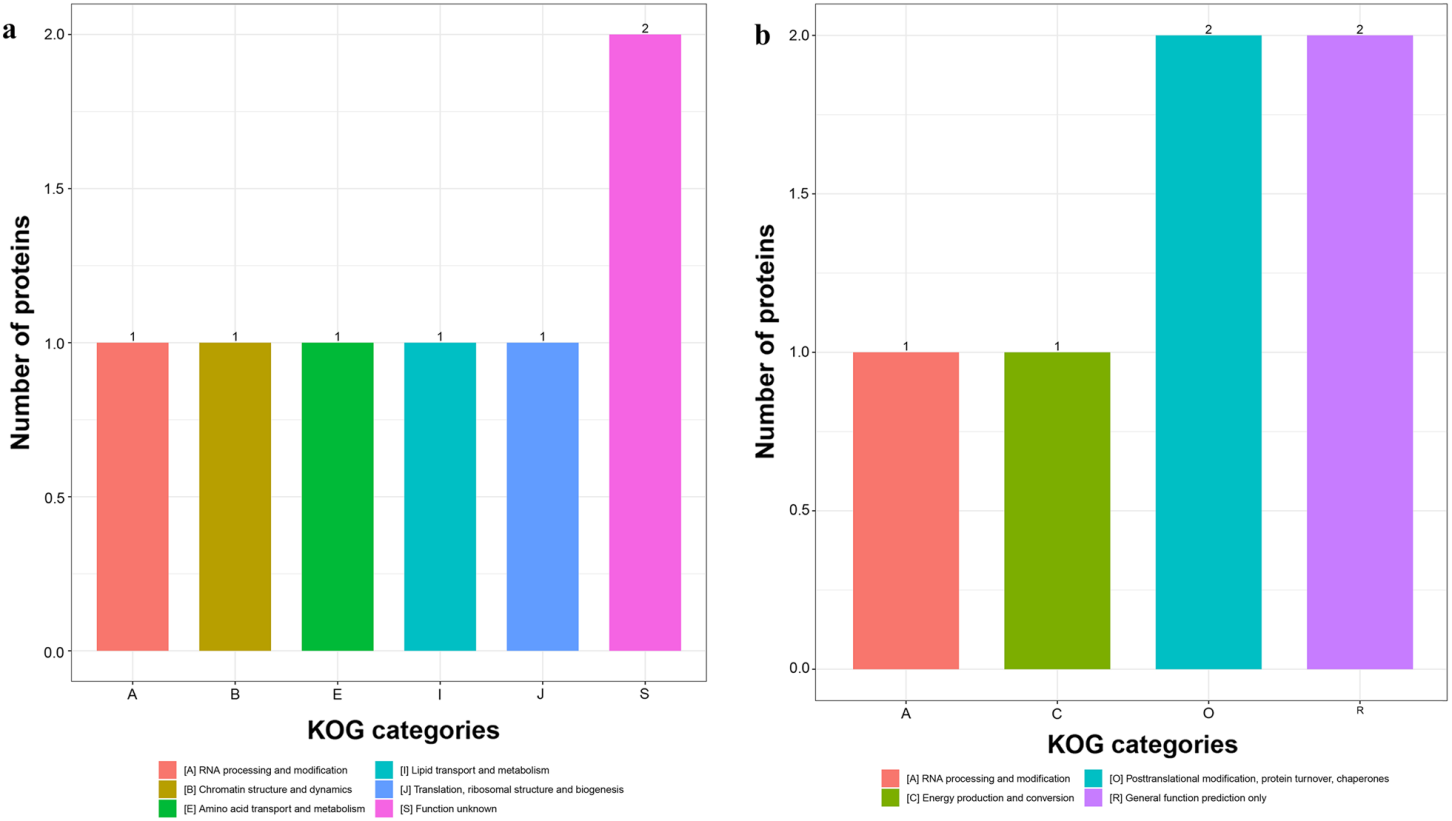
KOG functional classification chart of 17 differentially expressed proteins. **a** KOG enrichment of upregulated proteins. **b** KOG enrichment of downregulated proteins

For TMT quantification, raw datasets were used to calculate the ratios of the TMT reporter ion intensities in MS/MS spectra to obtain fold changes between samples. We used a 1.2-fold increase or decrease in protein expression as a benchmark for physiologically significant changes to determine which proteins were differentially expressed after excystation. In total, 17 DEPs were identified between excysted and non-excysted oocysts. Among these DEPs, 10 were upregulated and 7 were downregulated after excystation (Additional file 5: Table S3). According to the GO enrichment analysis (Fig. 4), 13, 8, and 12 proteins were enriched for biological process, cell component, and molecular function, respectively. Subcellular localization predictions showed that the DEPs were located in the cytoplasm (4), plasma membrane (3), nucleus (3), extracellular space (2), mitochondria (2), cytoplasm or nucleus (1), and cytoskeleton (1) (Fig. 5). Upregulated proteins were located in cytoplasm (5), mitochondria (2), nucleus (2), and plasma membrane (1). Downregulated proteins were located in the extracellular space (2), plasma membrane (2), cytoplasm (1), and nucleus. Functional enrichment analyses of the DEPs (Fig. 6) suggested that the DEPs were significantly enriched for “RNA processing and modification” (2), “posttranslational modification, protein turnover, chaperones” (2), “general function prediction only” (2), “chromatin structure and dynamics” (1), “energy production and conversion” (1), “amino acid transport and metabolism” (1), “lipid transport and metabolism” (1) and “translation, ribosomal structure, and biogenesis” (1). However, these proteins were not enriched in any KEGG pathways. In addition, we focused on upregulated proteins after excystation of *C. andersoni* oocysts. The unregulated proteins were significantly enriched in “binding” (3) and were mainly localized in the cytoplasm (3), nucleus (2), and mitochondria (2) (Fig. 6), suggesting that upregulated proteins were mainly associated with control of gene expression at the level of transcription, for example regulator of chromosome condensation 1 (RCC1), histone H2A, and an unidentified protein (ID: A0A1J4MP75).

### Validation of the transcription of DEPs by qRT-PCR

**Fig. 7:**
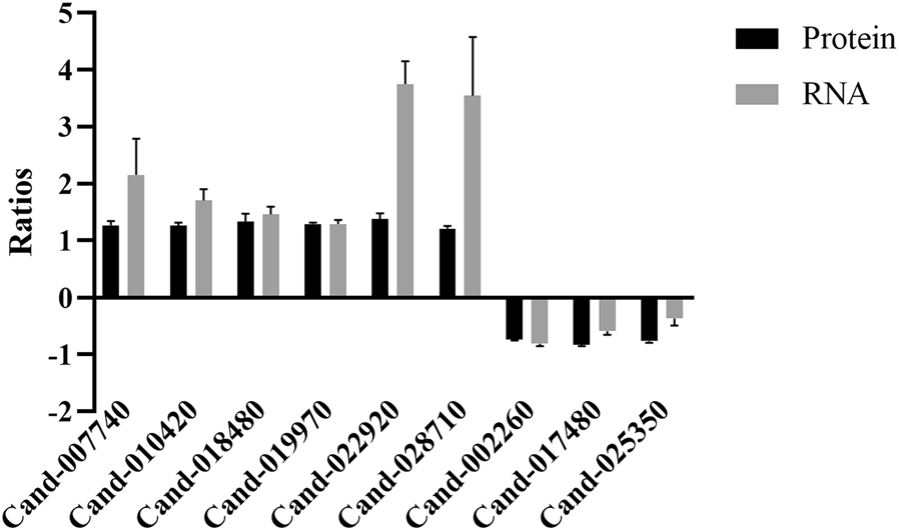
Transcriptional and proteome expression patterns of identical proteins/genes

To confirm the proteomic data, nine DEPs were selected randomly for qRT-PCR analysis to characterize gene expression. The qRT-PCR results indicated that the transcript levels of most genes were consistent with the protein levels based on TMT proteomics (Fig. 7).

## Discussion

To obtain a better understanding of the molecular mechanisms and proteome changes occurring when *C. andersoni* excysts in vitro, a TMT-based quantitative proteomics analysis was performed to directly screen differentially abundant proteins involved in excystation. We found that the number of identified proteins in the excystation of *C. andersoni* was greater than in previous proteomics studies of oocysts and sporozoites of *C. parvum*. In 2007, 303 proteins were identified during sporozoite excystation of *C. parvum* [14]. In 2008, 1237 nonredundant proteins (approximately 30% of the predicted proteome) of excysted *C. parvum* oocyst/sporozoite were identified using LC–MS/MS analysis [20]. In 2013, 33 separate *C. parvum* sporozoite proteins were identified from 135 protein hits using SDS-PAGE with subsequent LC–MS/MS analysis [21]. Among the proteins identified in this study, many have been identified as putative virulence factors by immunological and molecular methods [1]. For example, serine protease and aminopeptidase are associated with excystation; P23 and P30 are associated with adhesion; TRAP and thrombospondin-related proteins are involved in parasite gliding motility and cell penetration; Cp2, Cap135, and secretory phospholipase are associated with invasion, and HSP70 and HSP90 are associated with stress protection [1].

We used a comparative proteomics approach to identify 17 DEPs between the excysted and non-excysted *C. andersoni* oocysts. Analysis of these DEPs was performed to determine the mechanism of *C. andersoni* excystation, especially the upregulated proteins. Previous studies on the proteomics of excystation of *C. parvum* oocysts indicated that 26 proteins increased significantly during excystation [14]. The expression of Cap135 (contains LCCL domains) quadrupled within 30 min after excystation of the *C. parvum* oocyst, which may be involved in the adhesion process [14]. However, four proteins containing LCCL domains had no significant change in the present study, indicating that the role of those proteins may differ in *C. parvum* and *C. andersoni*. The expression of ribosomal protein increased during excystation of the *C. parvum* oocyst, which may be due to the rapid activation of protein synthesis after a variable period of oocyst quiescence [14]. Compared with non-excysted *C. andersoni* oocysts, RCC1 increased by a factor of about 3.5 in the excysted oocysts. Histone H2A and an unidentified protein (Protein accession: A0A1J4MP75) containing high mobility group box (HMG-box) domains were also increased after excystation. RCC1 is bound to chromatin and confined to the nucleus. The RCC1 ß-propeller domain binds the histone H2A/H2B dimer component of the histone octamer that can regulate the concentration gradient of Ras-related nuclear protein (Ran)-GTP around the chromosomes to mediate nucleocytoplasmic transport and mitotic spindle assembly [22–24]. An RCC1 mutant of *Toxoplasma gondii* showed defects in nuclear trafficking and growth impairment under nutrient limitation, demonstrating that the rate of nuclear transport is a critical factor affecting growth in low-nutrient conditions [25]. HMG-box proteins have been reported in many apicomplexan parasites, including *Plasmodium falciparum* [26], *T. gondii* [27], and *Babesia bovis* [28]. PfHMGB1 and PfHMGB2 are potent inducers of important mediators of inflammation, tumor necrosis factor alpha (TNFα) and inducible nitric oxide synthase (iNOS), suggesting that these proteins may have immunomodulatory roles in the pathophysiology of *P. falciparum* infection [26]. TgHMGB1 was implicated in transcriptional regulation and most likely acts as an activator of many virulence factors in *T. gondii* [27]. Based on these conclusions, we hypothesized that RCC1, H2A, and an unidentified protein were involved in the gene expression regulation of *C. andersoni* excystation factors.

Phosphatidylethanolamine (PtdEtn) had a 1.258-fold increase after excystation of *C. andersoni*. PtdEtn is located at the plasma membrane and is enriched in a variety of biosynthetic and metabolic processes, including lipid biosynthetic and metabolic processes, organophosphate biosynthesis and metabolism, phospholipid metabolism, and organic substance metabolism. The increase in PtdEtn may be associated with energy acquisition in the excystation of *C. andersoni* oocysts. PtdEtn is one of the most abundant phospholipids in prokaryotes and eukaryotes, and it contributes to membrane integrity, membrane fusion/fission, protein stabilization, and autophagy events [29]. PtdEtn is the second major phospholipid classified in *T. gondii*. Phosphatidylserine decarboxylase (PSD) mediates the decarboxylation of phosphatidylserine (PtdsSer) to form PtdEtn and displays much higher (tenfold) activity in *T. gondii* tachyzoites compared with yeast and mammalian cells [30]. In addition, choline kinase inhibitors inhibit the ethanolamine kinase activity of *P. falciparum* choline kinase, leading to a severe decrease in the phosphatidylethanolamine levels. This explains the resulting growth phenotype and parasite death [31].

The B6AJJ3 protein is an uncharacterized protein that contains a WD40-repeat-containing domain and a YVTN-type repeat domain. The expression level of the protein increased after excystation of *C. andersoni* oocysts by 1.345-fold. During the *Cryptosporidium* life-cycle, sporozoites slip out of the oocyst and infect the host cells, and this mainly occurs in the intestinal lumen and stomach. This uncharacterized protein may be associated with environmental stress and the host immune system. WD40 proteins are much more abundant in eukaryotic organisms, where they participate in a diverse set of functions, including signal transduction, cell division, cytoskeleton assembly, chemotaxis, and RNA processing [32]. The WD40-repeat protein-like protein PfWLP1 may support the stability of adhesion protein complexes of the blood stages of *Plasmodium* [33]. The YVTN-type repeat domain is found in archaeal surface layer proteins that protect cells from extreme environments [34].

In addition to the DEPs, many known and potential virulence factors were identified in the oocysts of *C. andersoni*. Although there were no significant differences in the expression of those virulence factors in the excysted and non-excysted *C. andersoni* oocysts, their role in sporozoite adhesion and invasion of host cells is still worthy of study. The Cpa135 protein was localized in the apical complex of the sporozoite and in the parasitophorous vacuole (PV) during the intracellular stages. In the oocyst–sporozoite, both the Cpa135 mRNA and the Cpa135 protein are present, and the protein rapidly increases during the excystation process of *C. parvum* [35]. This study identified a variety of heat shock proteins (HSPs), including HSP10, HSP70, and HSP90. HSPs are involved in maintaining cell homeostasis [36]. Synthesis of HSPs, especially HSP70, increases dramatically under stressful conditions such as a sudden temperature shift, changes in concentrations of glucose and calcium, or in response to immune effectors [37]. Previous studies identified two HSPs in *Cryptosporidium*, HSP70 and HSP90 [38, 39]. Differences in HSP expression in *T. gondii* correlate with parasite virulence in the immunocompetent host [40]. The relationship between the expression levels of these HSPs and *Cryptosporidium* virulence is worthy of further study.

## Conclusions

TMT-based proteomics technology provided a new method for the identification of proteins involved in the excystation of *C. andersoni* oocysts. The proteomes of excysted and non-excysted *C. andersoni* oocysts were compared, and multiple proteins including RCC1, histone H2A, and PtdEtn and two uncharacterized proteins (Protein Accession Numbers: B6AJJ3 and A0A1J4MP75) had increased expression in the excysted *C. andersoni* oocysts. These proteins may be key regulatory factors involved in the excystation of *C. andersoni*.

## Supplementary Information

**Additional file 1: Table S1.** Primers sequences designed for RT-qPCR.

**Additional file 2: Figure S1.** Sporozoites of *C. andersoni* observed by scanning electron microscopy.

**Additional file 3: Text S1. **Quality control report of protein extraction, labeling efficiency, and mass spectrometry stability.

**Additional file 4: Table S2.** Statistical summary of proteomic analyses and protein identification of *C. andersoni* oocysts before and after excystation.

**Additional file 5: Table S3.** Statistical analysis of differentially expressed proteins in *C. andersoni* oocysts before and after excystation.

## Data Availability

The datasets supporting the conclusions of this article are included within the article and its additional files. The mass spectrometry proteomics data have been deposited at the ProteomeXchange Consortium via the PRIDE partner repository with the dataset identifier PXD028423. All analyzed data are available from the corresponding author upon reasonable request.

## Abbreviations

TMT: Tandem mass tags
HPLC: High-performance liquid chromatography
LC–MS/MS: Liquid chromatography–tandem mass spectrometry
DEP: Differentially expressed protein
FDA: Food and Drug Administration
PBS: Phosphate-buffered saline
DIC: Differential interference contrast
SEM: Scanning electron microscopy
DTT: Dithiothreitol
CHAPS: 4% 3-[(3-Cholamidopropyl) dimethylammonio]-1-propanesulfonate
TEAB: Triethylammonium bicarbonate
FDR: False discovery rate
GO: Gene Ontology
KOG: Eukaryotic Orthologous Groups
KEGG: Kyoto Encyclopedia of Genes and Genomes
SDS-PAGE: Sodium dodecyl sulfate polyacrylamide gel electrophoresis
qRT-PCR: Quantitative real-time polymerase chain reaction
HCD: Higher-energy collision-induced dissociation
PV: Parasitophorous vacuole
HSPs: Heat shock proteins
