# Supplementary material for: Tandem mass tag (TMT)-based proteomic analysis of Cryptosporidium andersoni oocysts before and after excystation
Source: Parasit Vectors. 2021 Dec 18;14:608. doi: 10.1186/s13071-021-05113-6 (PMC8683822; doi:10.1186/s13071-021-05113-6)
Supplement: Supplementary file 1 — Additional file 1: Table S1. Primers sequences designed for RT-qPCR. [file 13071_2021_5113_MOESM1_ESM.docx]

Table S1. Primers sequences designed for RT-qPCR

| Gene Name | Protein description | Primer sequence (5'-3') | | No. of bp | |  |
| --- | --- | --- | --- | --- | --- | --- |
| **Cand_007740** | **40s ribosomal protein** | | F-AGCTTGGTCTCGGAAAGTTCGTT | | ~80 |  |
|  |  |  | R-TTTGAACCTATTCCGAGAGAGGC | |  |  |
| **Cand_019970** | **Uncharacterized protein** | | F-ATGTCAGCCCCTGAAAGGCA | | ~110 |  |
|  |  |  | R-ATCGGTTCCAACCAACCACA | |  |  |
| **Cand_022920** | **Histone H2A** | | F-AGGGATGCAAAGAAGACCCG | | ~117 |  |
|  |  |  | R-TGGAAGTACACCACCACTAGC | |  |  |
| **Cand_018480** | **Uncharacterized protein** | | F-CCGCTTGGATTACCTGTTGGA | | ~136 |  |
|  |  |  | R-GACGTTCAAATGCACGACGC | |  |  |
| **Cand_010420** | **Ethanolaminephosphotransferase** | | F-TGTGGTCTAGTGCTTGTTGGA | | ~143 | |
|  |  |  | R-CCAAGTCTTCTTGCTTGCTTACC | |  |  |
| **Cand_028710** | **Uncharacterized protein** | | F-ATCCAAGGGTTACCTGTTTCTGA | | ~146 |  |
|  |  |  | R-CCTAGTCCAGTTGAGAATCGACG | |  |  |
| Cand_025350 | Uncharacterized protein | F-ACTTGTTTGGAGTAGGCCGAG | | ~118 | |  |
|  |  | R-TGTTCCAACTGTCCAAGGATTAACA | |  |  |  |
| cand_002260 | Thioredoxin reductase | F-TGTTGGAGGGAAACCTAATGTTC | | ~146 | |  |
|  |  | R-ACCCAGCTGTTTCAAGTCCTAT | |  |  |  |
| cand_017480 | Aspartyl protease family protein | F-AGTTCCTGGAGCTGCACTAT | | ~187 | |  |
|  |  | R-ACCACGTTCAATATCTTCTTCTTGA | |  |  |  |
| 18S rRNA |  | F-GGAAGGGTTGTATTTATTAGATAAAG | | ~189 | |  |
|  |  | R-TCTCAGGCTCCCTCTCC | |  |  |  |

Note: Bold indicates upregulated protein
