# Supplementary figures and images for: Tandem mass tag (TMT)-based proteomic analysis of Cryptosporidium andersoni oocysts before and after excystation
Source: Parasit Vectors. 2021 Dec 18;14:608. doi: 10.1186/s13071-021-05113-6 (PMC8683822; doi:10.1186/s13071-021-05113-6)

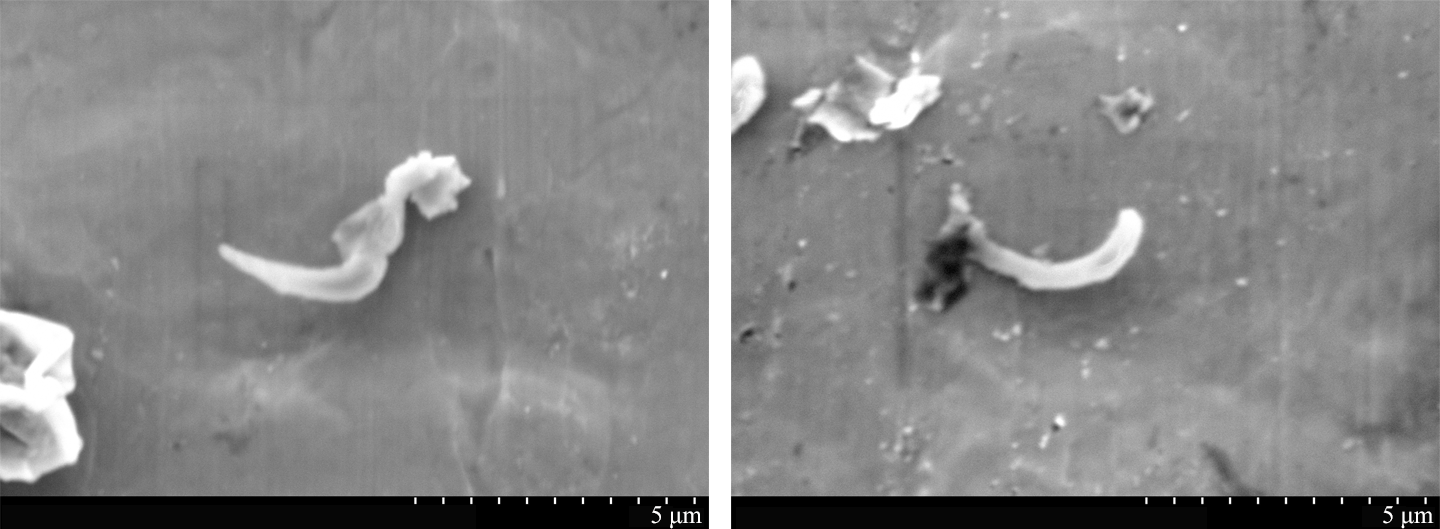

Supplement: Supplementary file 2 — Additional file 2: Figure S1. Sporozoites of C. andersoni observed by scanning electron microscopy. [file 13071_2021_5113_MOESM2_ESM.tif]
