# Supplementary material for: Tandem mass tag (TMT)-based proteomic analysis of Cryptosporidium andersoni oocysts before and after excystation
Source: Parasit Vectors. 2021 Dec 18;14:608. doi: 10.1186/s13071-021-05113-6 (PMC8683822; doi:10.1186/s13071-021-05113-6)
Supplement: Supplementary file 3 — Additional file 3: Text S1. Quality control report of protein extraction, labeling efficiency, and mass spectrometry stability. [file 13071_2021_5113_MOESM3_ESM.docx]

**Quality control report**

**1 Protein extraction quality control**

1.1 SDS-PAGE

Figure 1 SDS-PAGE (15 μg for each lane)

According to the quality judgment criteria of protein extraction, the protein bands were clear and uniform, the protein was not degraded, the swimming lanes in the group were parallel, the electrophoretic behavior was different between the groups, and the total protein could meet the requirements of two experiments.

1.2 Concentration determination result

Table 1. Results of protein concentration determination

| Sample number | Protein concentration (μg/μL) | volume of sample (μL) | Total protein (μg) |
| --- | --- | --- | --- |
| E1 | 2.0 | 750 | 1536.5 |
| E2 | 1.9 | 750 | 1415.4 |
| E3 | 2.3 | 750 | 1725.0 |
| P1 | 2.3 | 750 | 1726.9 |
| P2 | 2.4 | 750 | 1765.4 |
| P3 | 2.3 | 750 | 1753.8 |

**2 Evaluation of labeling efficiency and method stability**

2.1 Mark efficiency quality control

Table 2 Sample labeling information

| Sample number | Label |
| --- | --- |
| E1 | 126 |
| E2 | 127 |
| E3 | 128 |
| P1 | 129 |
| P2 | 130 |
| P3 | 131 |

Mark efficiency test results: The detection result of marking efficiency was 97.91%, which reached the quality control standard (>97%).

2.2 Methods Stability and quality control

In order to evaluate the accuracy, parallelism and difference of the quantitative method, we added an appropriate amount of standard proteins (GST and MBP) into each sample. The standard protein was added in trace amount and originated from prokaryotic cells, so it would not affect the quantitative results of protein in the sample. he standard protein addition rule is: 1) Add the same amount of standard protein to the samples repeated for many times; 2) Add 1:4:1:4... (M/M) differential standard protein in experimental group and compare groups of samples. The deviations of the two standard proteins were ±3.4% (GST) and ±5.4% (MBP) (quality control standard was ±20%), indicating that the accuracy and consistency of the quantitative method were good and the quality control standard was reached. In terms of difference, the mean quantitative results were 2.80 (GST) and 2.50 (MBP) times (the quality control standard was 2-4 times), indicating that the technical process could accurately reflect the sample difference.

**3 Mass spectrometry stability quality control**

Quality axis stability quality control standard: the central axis within ±3 PPM, the main section within ±10 PPM.

The central axis of the mass axis of this project is less than 1 PPM, and the main body is less than 5 PPM, indicating that the mass axis of the mass spectrum is accurate and stable.

Figure 2 Peptide mass error distribution diagram
